# The Immunophenotyping Changes of Peripheral CD4+ T Lymphocytes and Inflammatory Markers of Class III Obesity Subjects After Laparoscopic Gastric Sleeve Surgery – A Follow-Up Study

**DOI:** 10.2147/JIR.S282189

**Published:** 2021-05-05

**Authors:** Nasser M Rizk, Amina Fadel, Wasaif AlShammari, Noura Younes, Moataz Bashah

**Affiliations:** 1 Biomedical Sciences Department-College of Health Sciences, QU Health—Qatar University; 2 Biomedical Research Center, Qatar University; 3 Biomedical and Pharmaceutical Research Unit, QU Health—Qatar University; 4 Clinical Chemistry Lab, Hamad Medical Corporation, Doha, Qatar; 5 Metabolic Unit, Surgery Department, Hammed Medical Corporation (HMC), Doha, Qatar

**Keywords:** class III obesity, immunity, peripheral CD4+ T-lymphocytes, T-regulatory cells, follow up, laparoscope gastric sleeve surgery

## Abstract

**Purpose:**

Obesity is a chronic disorder characterized by a low-grade inflammatory state and immune cell irregularities. The study aimed to follow up on the changes in the peripheral CD4+ T lymphocytes and the pro-inflammatory cytokines; IL-6, TNF-alpha, MCP-1, and IL-10 at baseline and 12 weeks post-surgical intervention by the laparoscopic gastric sleeve (LGS) in morbidly obese patients (class III obesity subjects).

**Materials and Methods:**

A prospective longitudinal research included 24 class III obesity subjects with a BMI > 40 kg/m^2^. The subjects were enrolled from the Metabolic/Surgical Department at Hamad Medical Corporation (HMC)-Qatar. Fasting blood samples were collected at admission to LGS for weight loss and after 12 weeks of LGS. The immunophenotype of CD4+ T-cell populations; naïve (CD45RA^+^and CD27^+^), central memory T cells (CD45RO^+^ and CD27^+^), and effector memory (CD45RO^+^and CD27^−^) and T-regulatory cell (CD4+CD25+ FoxP3+) were identified using flow cytometry. Plasma pro-inflammatory cytokines and adipokines were evaluated. A control group of lean subjects was used to compare changes of T-regulatory and inflammatory biomarkers with postoperative changes in obese patients.

**Results:**

The means (SD) of age and BMI of class III obesity subjects was 32.32 (8.36) years and 49.02 (6.28) kg/m^2,^ respectively. LGS caused a significant reduction in BMI by 32%, p<0.0001. LGS intervention significantly decreased CD4+ T-lymphocytes and effector memory (TEM) cells but increased T-regulatory (Treg), naïve, and central memory (TCM) cells, with all *p* values < 0.05. The increase of Treg cells postoperative is significantly lower compared to lean subjects, p < 0.05. A significant reduction of plasma IL-6, TNF-α, and MCP-1, but IL-10 significantly increased after LGS, with all *p*<0.05. Adiponectin/leptin ratio improved after LGS by 2.9 folds, *p*<0.0001.

**Conclusion:**

Weight loss by LGS accomplished a substantial rise of Treg and decreased EM T-lymphocytes with a shift from pro-inflammatory to the anti-inflammatory pattern.

## Plain Summary

Obesity is a chronic disorder associated with comorbidities such as diabetes, hypertension, atherosclerosis, cardiovascular diseases, and cancers. Obese subjects with BMI ≥40kg/m^2^ are at high risk for morbidity and mortality, and nowadays, for COVID-19 infections. These obese subjects are treated nowadays with metabolic bariatric surgery, most commonly laparoscopic gastric sleeve (LGS) surgery, which showed marked success. The current study investigated the profile of circulating immune system CD4+ cell subpopulations at baseline and 12 weeks after LGS in these patients. The results demonstrated a shift of immune cells of CD4+ lymphocytes towards upregulation of T regulatory cells, naïve, central memory cells with downregulation of effective memory cells. LGS alleviated the obesity-associated inflammation and insulin resistant states associated with morbid obesity.

## Introduction

Obesity is a chronic disease associated with inflammatory responses in adipose tissue associated with metabolic and vascular abnormalities, such as insulin resistance, fatty liver, arteriosclerosis, and cardiovascular risks.^1^ Obesity is increasing worldwide in prevalence among the developing and developed countries, which need medical management to prevent its associated comorbidities.^2^ Although class III obesity, especially with BMI>40 Kg/m^2^, is associated with several comorbidities, it can be controlled, and the consequences could be prevented.^3^ Nowadays, successful weight loss intervention for class III obesity (BMI >40 Kg/m^2^) with significant comorbidities is the metabolic/bariatric surgery, which is one of the most effective interventions compared to other management and has proved as effective therapy with excellent results.^4^

Previous studies demonstrated that inflamed adipose tissue in obese mice was associated with increased macrophages and T lymphocytes. The infiltration of those immune cells into the adipose tissue most likely causing the inflammation.^5^ Previous studies also suggested that the increased number of inflammatory cells and their secretions such as cytokines induced an increased rate in differentiation and proliferation of adipocytes followed by fat accumulation, obesity, insulin resistance, and increased risks to cardiometabolic syndrome.^6^

The T lymphocytes in visceral adipose tissue (VAT) of obese rodents and humans were postulated to play a role in obesity-provoked inflammation.^7^ Several studies demonstrated that in the diet-induced obese mice by a high-fat diet, the increased quantities of T lymphocytes are mostly preceding the macrophage infiltration in VAT and playing significant roles in the recruitment of the macrophage recruitment and induction of VAT inflammation.^8^,^9^

Regulatory T (Treg) cell is one of the main regulatory subsets among CD4^+^ T lymphocytes, characterized by a specific molecular marker as CD4^+^ CD25^+^ Foxp3^+^. Although several effector T cell subcategories are involved in adipose tissue inflammation, Treg cells are concerned with maintaining the AT homeostasis.^10^

Moreover, a previous study of CD4+T lymphocytes in Class III obese patients’ blood demonstrated an increase in peripheral blood CD4^+^ T Cells with a shift toward natural CD4^+^CD25^+^FoxP3^+^ T-regulatory associated with anti-inflammatory profile with the increased plasma level of cytokines IL-7 and CCL5.^3^

The cardiometabolic indorsing of obesity is not only local changes of AT microenvironment but also systematically through the dysregulated profile of the circulating immune cells and inflammatory biomarkers that could reflect adipose tissue pathology such as inflammation. However, no data are available concerning the alterations in circulating CD4+ T-lymphocyte immunophenotyping with class III obese subjects after LGS intervention. The present study’s main aim is to investigate the alterations of the immune phenotype of CD4+ T-lymphocyte subpopulations in class III obesity undertaking bariatric surgery and after 12 weeks of the intervention. The study also investigated parallel changes of adipokines, metabolic, and inflammatory markers at baseline and after 12 weeks of LGS.

## Materials and Methods

### Study Design

A prospective longitudinal study was performed on a total of 24 class III obesity subjects with BMI of (38.8–64.0 kg/m^2^) of both sex (15 females and 9 males) with age ranges between 10 and 38 years in the Metabolic/Surgical Department at Hamad Medical Corporation (HMC). Another healthy control group (n=28) with a BMI of (32.91 ± 6.67 kg/m^2^) was used to compare healthy and class III obesity groups. The healthy control group was recruited from students and employees at Qatar University with BMI from 18.50–24.00 and from 30.00 to 34.99 kg/m^2^, respectively. Among the cohort class III obesity study subjects with BMI ≥40 kg/m^2^ at the baseline, eight subjects were diabetic (33.3%) based on diagnostic criteria of ADA,^11^ and eleven subjects had metabolic syndrome (45.8%) according to ATP III.^12^ Insulin resistance was calculated by HOMA-IR,^13^ with 4.8 as a cut-off point (75th percentile).^14^ All subjects included in the current study were excluded if they had the immunologic/autoimmune disorder, severe renal and liver impairment, thyroid disorders, and cancer. Venous blood samples (10 mL) were collected from all consented subjects at admission to surgery consecutively performed laparoscopic gastric sleeve (LGS) operation for weight loss by authorized consultants surgeons at Bariatric Metabolic Surgery, HMC, Doha-Qatar. Another blood specimen was obtained after three months of follow up. A complete blood picture was assessed at HMC labs. Plasma samples were used for biochemical analysis, including circulating levels of glucose, lipid profile, liver, and kidney functions, hormonal parameters (c-peptide, insulin, leptin, and adiponectin), hsCRP, and blood cell immune markers (CD4^+^ T lymphocyte (Treg, naïve, central memory [TCM], and effector memory [TEM]) and pro-inflammatory cytokines and chemokine [MCP-1, TNF-alpha, IL-6, and IL-10]. Adipose tissue biopsy from visceral white adipose tissue (VAT) (1g) was collected during LGS surgery (first visit) and was used to count the adipose tissue macrophages to characterize the M1/M2 polarization. All CD markers and flow cytometry reagents used for immunophenotyping were purchased from (BD, Bioscience, USA) unless otherwise mentioned (Table S1). Multiplex Elisa kits from (Millipore, Merck, Germany) were used to measure adipokines and inflammatory markers. Other chemicals were obtained from (Sigma-Aldrich, Germany) unless mentioned elsewhere. The study was approved by the Institutional Review Boards of the Hamad Medical Corporation and Qatar University (HMC # 12199/12, and QU-IRB# 279-A-2104 for class III obesity subjects, and QU-IRB#683-EA/116 for the control group, respectively). After a full explanation of the purpose, nature, and risk of all procedures used, written informed consent was taken from all subjects for acceptance in this study. The study was accomplished according to the principles stated in the Declaration of Helsinki.

### Blood Collection and Assays

A total of 10 mL of venous blood was obtained from a peripheral vein and was subjected for centrifugation and subsequent plasma separation and eventual central storage at −80°C for further analysis of MCP-1, TNF-alpha, IL-6, leptin, adiponectin, and IL-10. Another 10 mL of the venous blood sample was collected into EDTA tube and stored at room temperature. It was used for determining the total and differential leukocyte count (TLC and DLC), CD4+ T-lymphocytes, and its populations and cell immune markers (CD4^+^ T cell and its subpopulations) using flow cytometry. Plasma samples were used for biochemical analysis, including glucose, lipid profile, liver, kidney functions, hormonal parameters (c-peptide and insulin), and were performed at HMC labs. Assessment of MCP-1, TNF-alpha, IL-6, leptin, adiponectin, and IL-10 was performed in the Biomedical Research Center (BRC) labs at Qatar University using Luminex 200 by the multiplex technique according to the manufacturer’s protocol. Adipokines and cytokines were measured by the Multiplex Elisa technique using magnetic beads (HADK2MAG-61K, MADCYMAG-72K from Millipore), IL-10 (MPXHCYTO-60K-06 from Millipore), and Elisa was used for hsCRP (ab181416 from Abcam). The assay was performed based upon the manufacturer’s protocol, as previously published by our lab.^15^

### Flow Cytometry

Staining and procedures were accomplished according to the manufacturer’s protocol. In short, blood specimens were collected on EDTA tube and stored at a constant temperature of 4°C, and analyzed within two hours of collection. For immunophenotyping CD4+ T-cell population cells, the circulating CD 45+/CD4 was counted using (BD LSRFortessa TM Cell Analyzer cat no. 649225, BD FACSDiva Software), which acquired at least 5×10^5^ events, Supplementary Figures. CD4+ T-cell subpopulations were defined as naïve (CD45RA^+^andCD27^+^), central memory T cells (CD45RO^+^andCD27^+^), effector/peripheral memory (CD45RO^+^and CD27^−^) and Treg cells were defined as CD4+CD25+FoxP3+, as previously published.^3^,^16^ Briefly, 100µL blood was incubated with 10µL PerCP-conjugated anti-human CD45 monoclonal antibody (Becton Dickinson Biosciences), 10µL PerCP-conjugated anti-human CD4 monoclonal antibody APC (Becton Dickinson Biosciences, USA), and 10µL allophycocyanin-conjugated anti-human CD27 APC (Becton Dickinson Biosciences, CA, USA) for 30 min at 4°C.^3^,^16^,^17^ Briefly, 100µL blood was incubated with 10µL PerCP-conjugated anti-human CD45 monoclonal antibody (Becton Dickinson Biosciences), 10µL PerCP-conjugated anti-human CD4 monoclonal antibody APC (Becton Dickinson Biosciences, USA), and 10µL allophycocyanin-conjugated anti-human CD27 APC (Becton Dickinson Biosciences, CA, USA) for 30 min at 4°C. The number of peripheral blood cells, which was positive for CD markers, was decided by a three-dimensional fluorescence dot-plot analysis of the samples, following the proper gating. We primarily gated CD45+ peripheral blood cells, then gating with 7-AAD for viability, then gated for CD4+ blood cells in the lymphocyte cell section, and afterward analyzed the resulting population for expression of CD27 (Figure S1). Data were handled using a software program (BD FACSDiva Software, BD Bioscience). Cell counts were presented as the number of cells per 10^6^ cytometric events and displayed as a percentage of total CD4+ T lymphocytes. The technician performed all assays were blinded to the medical condition of participants. For detecting FoxP3 T regulatory cell, in a tube containing 100-μL of the buffy coat, a 20-μL from each reagent (BD-PE CD25 and BD-FITC CD4) was added and incubated for 20 minutes at room temperature (RT) in the dark. After that, 1 mL of “PE Pharmingen Stain Buffer (FBS)” was added to the tubes and centrifuged for 10 minutes at 1500 rpm. A clear pallet was observed at the bottom of the tube; the supernatant FBS was aspirated. Then, while adding 1 mL of “PE-Human FoxP3 Buffer A”, the pellet was resuspended gently; then incubate the tubes for 10 minutes in the dark at RT. Following that, the sample was centrifuged at 3000 rpm for 5 minutes. The supernatant was decanted, and 0.5 mL of buffer C was added to tubes followed by tube incubation. Buffer C is a mixture of “60-μL of PE-Human FoxP3 Buffer A and 940-μL of PE-Human FoxP3 Buffer B”. Repeating the staining “FBS” step, 20-μL was added of PE-Mouse anti-Human Foxp3 antibody and incubated in the dark for 30 minutes at RT. Finally, 500-μL of FBS was added, and the results were analyzed using a flow cytometer. The process of flow cytometry was based on previous publications,^3^,^16^,^17^ as shown in Supplementary Figures (Figure S2).

### Stromal Vascular Isolation and at Macrophages

Visceral adipose tissues were obtained from the omentum during LGS and placed immediately in DMEM supplemented with 20 mmol/l HEPES (Sigma) and transferred to the lab within 2 h. Upon receiving the sample, adipose tissue was weighed and minced using razors, scissors, and tongs in the Biosafety level 2 hoods. It was then washed with PBS+1% antibiotic wash buffer. Warmed Type 1 Collagenase is then added according to the tissue’s weight, and tubes were covered with parafilm and placed in the shaking water bath for 60 minutes. After digestion, the sample is centrifuged to get a pellet of Stromal-Vascular (SV) cells with a transparent lipid layer on the top. The lipid layer is removed, and the pellet is washed twice with warmed PBS+1%BSA. The RBC lysis solution is added, and the sample is centrifuged after 10 mins. The supernatant is then removed, and the sample is mixed thoroughly with stromal medium and filtered through a (190 µM) sieve filter. The filtrate was then resuspended in one mL stromal medium and processed for flow cytometry. Cell number and viability were assayed using a hemocytometer and trypan blue. The flow cytometry process was based on previous publications using CD206 and CD11b markers to identify M1/M2 polarization, as previously published.^18^

### Statistical Analysis

The expression of data is presented as mean ± SD unless otherwise mentioned. All data were checked for outliers, skewness, and normality and transformed when necessary. We compared baseline parameters between class 111 obese subjects before and after surgery using appropriately paired student-t or Mann–Whitney/Wilcoxon (if the normality assumption was violated). One-way analysis of variance or Wilcoxon test (if the normality assumption was violated) to compare parameters between three groups followed by Dunn's test for mutiple compariosns . The Chi-square test evaluated the distribution of categorical variables. Spearman correlation coefficient (r) was applied to study the study variables’ correlations with the immune cells and BMI. The cut-off value for significance is a two-tailed level p < 0.05. All analysis was achieved by employing the SPSS program for Windows (version 23 statistical software; Texas instruments, IL, USA), and Graph Pad Prism was utilized to draw the figures (version 8, for Win, Graph Pad Software, La Jolla California USA).

## Results

### Effect of LGS Surgery on Anthropometric, Clinical, and Biochemical Characters of the Morbidly Obese Subjects

A clinical cohort of 24 morbidly obese subjects was recruited. The cohort had a range of ages from 19 to 48 years, with BMI, ranges from 38.8 to 64.0 kg/m^2^. Participants were evaluated for blood pressure, glucose, insulin, lipid profile, adipokines, and inflammatory markers and followed up after 12 weeks of LGS. Liver and renal functions were evaluated; aspartate transaminase (AST) was 34.64 (16.44)U/L, and alanine transaminase (ALT) was 31.72 (27.33) U/L, and creatinine was 60.91 (10.54) μmol/L. Cortisol was evaluated to exclude endocrine disorders, and its concertation was 301.75 (134.04) nmol/l. Initially, 33.3% of morbidly obese subjects had type 2 DM (T2DM), and 67.0% had fasting hyperglycemia. Among the cohort, 29.0% had dyslipidemia, 90.0% had increased WC above cut off values, and 33.0% had hypertension. Among the cohort study, 62.5% had insulin resistance (IR), and the percentage of metabolic syndrome (MeS) was 54.0%. Table 1 describes the clinical and biochemical data of the study subjects. Females were more frequent in this study, 62.5% than the male gender. After LGS surgery, the study subjects’ follow-up revealed that body weight, BMI (Table 1), and waist circumference were significantly reduced by 36.0%, 32.6%, and 10.0% of the initial measurements, respectively, with *p*<0.0001. Of note, all biochemical parameters of glucose homeostasis, such as glucose, HbA1C, insulin, and insulin resistance, were significantly reduced after LGS. A significant reduction of TG and LDL while increasing HDL-C post-LCS surgery was detected, *p*< 0.05. Further, LGS surgery significantly reduced the percentage of metabolic syndrome components among the cohort subjects; WC from (92.0% to 67.0%); dyslipidemia from (29.0% to 14.0%); hyperglycemia from (75.0% to 37.5%), HOMA-IR from (62.5% to 45.8%), diabetes from (33.3% to 25.0%) and hypertension from (33.0% to 4.0%), all with (*p*<0.05). We also compared the demographic and biochemical data with healthy control subjects of lean and obese subjects to illustrate the changes after the LGS of class III obesity subjects. Furthermore, we investigated if the clinical and metabolic changes after LGS of class 111 obesity could improve; therefore, we recruited healthy lean and obese subjects as control groups for comparison. Table 2 shows that the demographic, clinical, and biochemical parameters were compared in healthy lean and obese subjects with the class III obesity group after 12 weeks post-LGS. The data showed that BW, BMI, and WC were significantly different between healthy lean controls (*P*≤0.05), while these parameters were not significantly different with the healthy obese subjects (*P*>0.05). Meanwhile, the following parameters, WC; TC; TG; LDL; insulin; HOMA-IR; leptin; MCP-1; and IL-6 (*P*≤0.05), were significantly higher in the class III obesity group after LGS than lean subjects, while IL-10 showed opposite changes. However, class III obesity group had significantly higher values for TC, insulin, and HOMA-IR than healthy obese subjects (*P*≤0.05), while IL-10 showed opposite changes.

**Table 1:** The Clinical, Hormonal, and Metabolic Characteristics of Study Subjects at Baseline and After 12 Weeks of the Laparoscopic Sleeve Gastrectomy (LGS)

Variables	Basal State (24) Before LGS	3 Months (24) After LGS	*P* value
Age (years)	32.32 (8.36)	32.45 (8.50)	0.867
Sex F/M (n, %)	15 (62.5%)/9 (37.5%)	15 (62.5%)/9 (37.5%)	0.859
Weight (kg)	136.56 (23.52)	87.38(15.94)	< 0.0001
BMI (kg/m^2^)	49.02 (6.28)	33.06(4.68)	< 0.0001
Waist circumference (cm)	121.16 (21.48)	109.00(21.38)	< 0.0001
SBP (mmHg)	117.17 (12.61)	115.21 (11.10)	0.261
DBP (mmHg)	80.72 (9.28)	78.12 (8.35)	0.247
Glucose (mM)	6.33 (1.14)	5.75 (1.04)	0.022
Insulin (mIU/l)	26.71 (12.41)	18.75 (11.95)	0.005
C-peptide (ng/mL)	3.09 (0.84)	2.7105 (0.75)	0.030
HbA1C%	6.47 (1.14)	6.09 (1.01)	0.005
TC (mM)	5.17 (1.06)	5.08 (1.12)	0.061
TG (mM)	1.28 (0.51)	1.15 (0.48)	0.004
HDL-C (mM)	1.12 (0.38)	1.25 (0.43)	0.050
LDL-C (mM)	3.51 (0.97)	3.37 (0.99)	< 0.0001
HOMA-IR	6.09 (2.64)	4.02 (2.45)	< 0.0001

**Notes:** Data are presented as mean and (SD) for the continuous data and numbers and (%) for categorical data. Two-tailed *p* value is significant at ≤0.05.

**Abbreviations:** BMI, body mass index; WC, waist circumference; SBP, systolic blood pressure; DBP, diastolic blood pressure; HbA1C, hemoglobin A1c; TC, total cholesterol; TG, triglycerides; HDL-C, high-density lipoprotein cholesterol; LDL-C, low-density lipoprotein cholesterol; HOMA-IR, homeostatic model assessment of insulin resistance.

**Table 2:** Comparison Between Healthy Control Subjects at Baseline and Morbidly Obese Subjects Before and After LGS

Variable	Healthy Control		Class III Obesity
	Lean (n=14)	Obese (n=14)	After LGS (n=24)
Age (years)	25.6 ± 6.55	32.38 ± 8.21	32.32±8.36
Weight (kg)	56.00 ± 2.65	97.02 ± 18.05	87.38± 15.94^* Ø^
Height (cm)	167.34 ± 4.27	161.48 ± 17.11	164.29 ±9.83
BMI (kg/m^2^)	20.04 ± 1.07	34.21 ± 3.29	33.06±4.68*
SBP (mmHg)	114.00 ± 6.96	118.33 ± 5.51	115.21± 11.10
DBP (mmHg)	83.6 ± 2.30	81.25 ± 8.27	78.12±8.35
Waist Circumference (cm)	74.6 ± 0.55	107.63 ± 6.47^∞^	109.00± 21.38*
Glucose (mM)	4.16±0.16	4.99±0.61	5.75± 1.04
Insulin (mIU/l)	6.55 ± 3.33	7.76 ± 6.93	18.75 ±11.95 ^* Ø^
TC (mM)	3.27± 2.13	3.37 ±2.19	5.08± 1.12^* Ø^
TG (mM)	0.93±0.15	1.17±0.29^∞^	1.15 ±0.48*
HDL-C (mM)	1.21±0.19	1.14±0.34	1.25 ±0.43
LDL-C (mM)	1.62±0.42	2.20±0.35^∞^	3.37±0.99^* Ø^
HOMA-IR	1.45± 1.33	1.49 ± 0.85	4.02 ±2.45^* Ø^
Il-10 (pg/mL)	3.77 ± 1.30	2.92 ± 0.63	0.74±0.32^* Ø^
Leptin (ng/mL)	8.21±4.45	25.62±6.34^∞^	18.34±5.84*
Il-6 (pg/mL)	0.74 ± 0.21	1.16 ± 0.57^∞^	1.11±0.53*
MCP-1 (pg/mL)	96.96 ± 5.30	141.85 ± 81.64	261.6±27.92^* Ø^

**Notes:** Data are presented as mean and (SD) for the continuous data and numbers and (%) for categorical data. The two-tailed p value is significant at ≤0.05. *P value is significant between Class III obesity subjects after LGS and lean subjects. ^Ø^ P value is significant between Class III obesity subjects after LGS and obese healthy subjects. ^∞^P value is significant between obese healthy subjects and lean subjects.

**Abbreviations:** BMI, body mass index; WC, waist circumference; SBP, systolic blood pressure; DBP, diastolic blood pressure; HbA1C, hemoglobin A1c; TC, total cholesterol; TG, triglycerides; HDL-C, high-density lipoprotein cholesterol; LDL-C, low-density lipoprotein cholesterol; HOMA-IR, homeostatic model assessment of insulin resistance; IL, interleukin.

**Figure 1:**
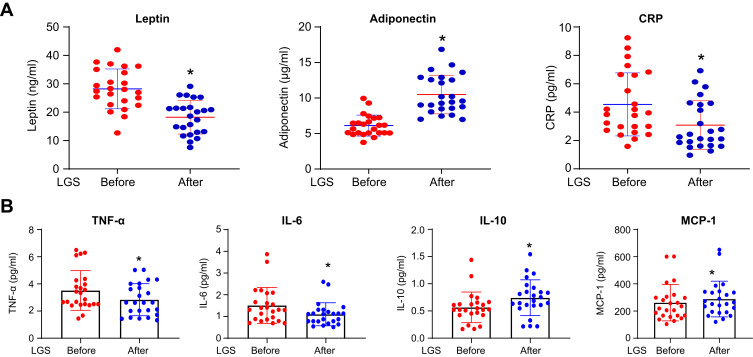
Adipokines, hsCRP, and cytokines change after 12 weeks of LGS. (**A**) Scatter dot plot represents mean and SEM of leptin, adiponectin, and hsCRP, before and after LGS, in the class III obesity group. *p value (after LGS) is significantly different than (before LGS). Two-tailed *p* value is significant at ≤0.05. (**B**) The scatter dot plot represents the mean and SEM of TNF-α-α, IL-6, IL-10, MCP-1 before (baseline) and after LGS in the class III obesity group. *p value (after LGS) is significantly different than (before LGS). Two-tailed *p* value is significant at ≤0.05.

### Effect of LGS Surgery on Circulating Concentrations of Adipokines, hsCRP Pro-Inflammatory Cytokines, and Chemokines in Class III Obesity Group

To gain more about the effect of LGS on biomarkers related to obesity and inflammation, we evaluated the effect of LGS on the concentrations of the following biomarkers in the plasma; acute phase protein (hsCRP), adipokines (leptin and adiponectin), pro-inflammatory cytokines (TNF-α, IL-6, and IL-10) and chemokine (MCP-1). Figure 1A, displays a significant reduction of leptin (Mean and SEM in ng/mL) from pre-LGS of 28.20 (7.02) compared to post-LGS of 18.23 (5.94), *p*< 0.0001, and hsCRP (pg/mL) from pre-LGS level of 4.62 (1.22) to post-LGS of 3.14 (1.17), *p*=0.002. On the contrary, LGS significantly increased the plasma concentrations of adiponectin (µg/mL) from pre-LGS of 6.12 (1.48) to post-LGS of 10.49 (2.71), *p*< 0.0001. Moreover, adiponectin/leptin ratio increased after LGS by 2.9 folds from 0.23±0.09 at pre-LGS to 0.67±0.39 after LGS, *p*< 0.0001. Figure 1B, demonstrated significant changes of the markers from pre-LGS to post-LGS of M1 polarization including, TNF-α from (3.51±0.29 to 2.84±0.24 pg/mL), IL-6 from (1.51±0.16 to 1.10±0.10 pg/mL) and MCP-1 (288.3±26.8 to 261.5±27.3 pg/mL) with *p*=0.021, *p*<0.0001, and *p*=0.012, respectively. IL-10, a marker of M2 polarization, significantly increased from (0.56±0.05 to 0.74±0.06 pg/mL) after LGS with *p*<0.0001. Therefore, the data showed a shift from M1 to M2 polarization with enhanced adiponectin/leptin ratio, reflecting a systemic improvement of adipose tissue inflammation.

### Adipose Tissue Macrophages in Obese Subjects

**Figure 2:**
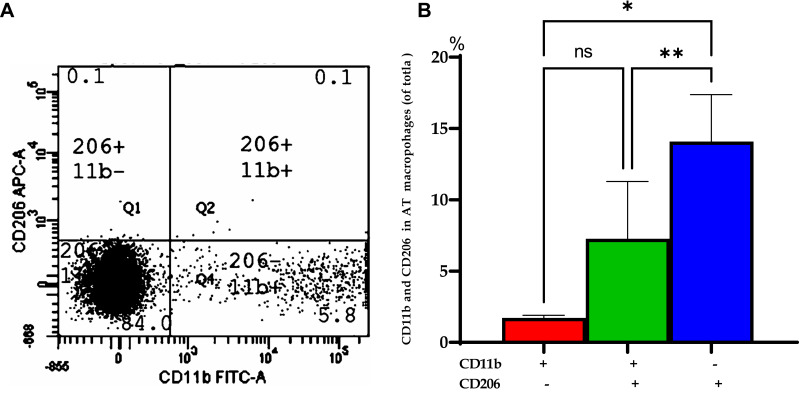
Distribution of CD206/CD 11b in visceral adipose tissue (VAT) of obese subjects at baseline. (**A**) A representative of flow cytometry for visceral adipose tissue (VAT) macrophages expressing CD206 and CD11b cells with percentages in different regions of the class III obesity group. (**B**) Bars represent the mean and SEM of VAT macrophages based on the expression of CD11b+ (M1) and CD206+ (M2). *p value for (CD206-, CD11b+) is significantly different than (CD206+, CD11b-). **p value for (CD206+, CD11b-) is significantly different than (CD11b+ and CD206+). ns: means no significant difference was observed between (CD11b+ and CD206+) and (CD206-, CD11b+). Two-tailed *p-*value is significant at ≤0.05.

To demonstrate the macrophage polarization M1 and M2 in the adipose tissue, the distribution of CD206/CD11b+ was evaluated in VAT of class III obesity subjects at baseline obtained during LGS. Of note, the expression of CD11b+ macrophages expression (M1 marker) was significantly higher by ≈8.3 folds than CD206+ (M2 marker) with *p*=0.003 while mixed (CD11b+ and CD206+) were significantly higher by 4.3 folds than CD206+ (M2 marker), *p*=0.031 as shown in Figure 2.

### Effect of LGS on Total White Blood Cell Count

**Figure 3:**
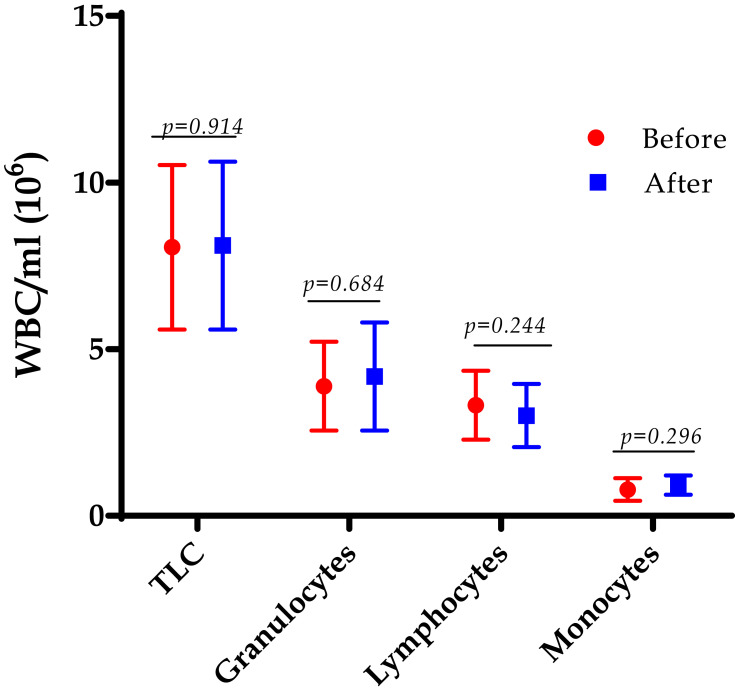
The total leukocyte count (TLC) and the differential count for granulocytes, lymphocytes, and monocytes were counted at the baseline (before) and after 12 weeks of the LGS in the class III obesity group. Data are presented as mean and (SD). Two-tailed *p-*value is significant at ≤0.05.

Total white blood cell count showed a non-significant difference in the total count and differential count of granulocytes, lymphocytes, and monocytes before and after LGS, though a slight decline was observed among lymphocytes *p*=0.244, as shown in Figure 3.

### Effect of LGS on CD4+ Lymphocytes and Subpopulations

**Figure 4:**
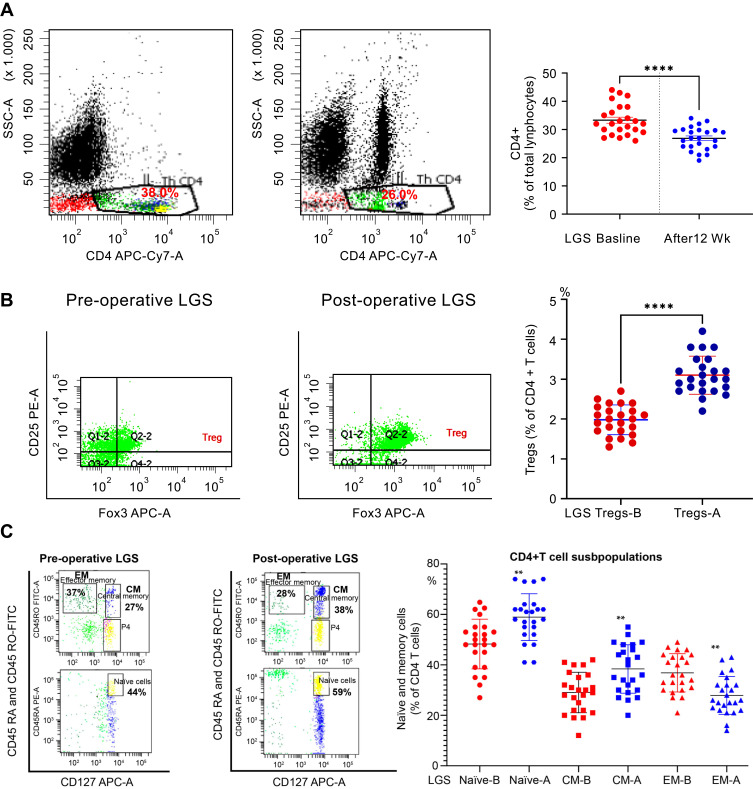
The CD4+ T lymphocytes at baseline and 12 weeks after the LGS. (**A**) A representative of flow cytometry for CD4+ T lymphocytes (CD4‏+CD45+‏‏) before and after LGS (left panel). Bars represent mean, and SD of Treg cells, and ****p value (< 0.0001) is significant after 12 weeks of LGS compared to initial bassline levels in class III obesity subjects. Two-tailed *p* value is significant at ≤0.05. (**B**) T regulatory CD4+ t cells count in class III obesity subjects before and after 12 weeks of LGS. A representative of flow cytometry for Treg cells (CD4‏+CD25+‏ Foxp3+‏) before and after LGS (left panel) in class III obesity group. Bars represent mean, and SD of Treg cells, and ****p value (< 0.0001) is significant after LGS compared to initial baseline levels. Two-tailed *p* value is significant at ≤0.05. (**C**) Naïve and memory CD4+ T cells count in morbidly obese subjects before and after LGS. A representative of flow cytometry for naïve and memory CD4+ t cells (left panel). Bars represent mean and SD of naïve, central memory (CM) memory, and effector memory (EM) CD4+ T cells. **p value is significant after LGS compared to initial baseline levels in the class III obesity group. Two-tailed *p* value is significant at ≤0.05.

**Abbreviations:** B, before LGS surgery at baseline; A, after 12 weeks of LGS surgery.

Figure 4 displays the effect of LGS on the CD4+ T-lymphocytes and its subpopulations of T regulatory, naïve, effector memory (TEM) and central memory (TCM) cells by flow cytometry on isolated polymorphonuclear leukocytes (PMNLs). LGS significantly decreased the total CD4+ lymphocytes from pre-LGS of 33.3±5.3% to post-LGS of 26.9±4.1%, *p*< 0.0001 (Figure 4A). LGS significantly induced the Tregs cell count post-LGS expressed as a percentage of total CD4+ cells (3.1±0.4%) compared to pre-LGS (1.9±0.3%) at baseline, *p*<0.0001 (Figure 4B). Furthermore, counting in the percentage of the CD4+T-cells subpopulations to the total CD4+ T cells by flow cytometry on isolated PMNLs revealed that LGS significantly induced the naïve cell by 1.22 folds from pre-LGS (48.3±9.3%) to post-LGS (58.9±9.2%), *p*< 0.0001, and central memory (TCM) cells increased by 1.32 folds from pre-LGS (29.0±8.0%) to post-LGS (38.4±9.6%), *p*< 0.0001, but effective memory (TEM) cells decreased by 1.31 folds from pre-LGS (36.8±7.6%) to post-LGS (27.9±7.5%), *p*< 0.0001 among the cohort of class III obesity subjects, as shown in Figure 4C. Further, we also observed the Tregs cell count post-LGS expressed as a percentage of total CD4+ cells (3.1±0.4%) compared to healthy control subjects in lean (7.07±2.49%), *p*< 0.0001 and in healthy obese subjects (4.67±1.63%), *p*= 0.094 – See Supplementary Figures (S4). Further, we explored the impact of insulin resistance and diabetes on T-reg cells at baseline and after LGS of class III obesity subjects, and results showed no significant difference at post-LGS was observed in obese subjects with andwithout both states of insulin resistance and diabetes, p>0.05, See Supplementary Figures (S5–S7).

### The Relationship Between Treg Cells and BMI, Metabolic Profiles, and Pro-Inflammatory Cytokines in Class III Obesity Group

Further, we evaluated the Spearman’s coefficient correlations (r) between CD4+Treg cells before (B) after (A) of LGS, and CD4+ subpopulations at the baseline of naïve, effector memory (TEM), and central memory (TCM) at baseline with metabolic parameters, adipokines, and chemokines, pro-inflammatory cytokines in class III obesity group, as shown in Supplementary Tables and Figures (Tables S3, S4 and Figure S8).

**Figure 5:**
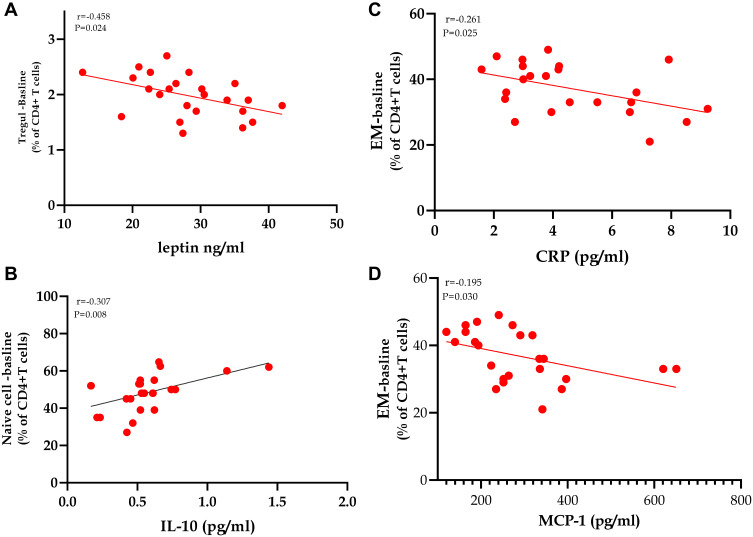
Spearman’s coefficient correlations between CD4+ T lymphocytes’ subpopulations with biomarkers of adiposity and inflammation in class III obesity subjects. (**A**–**D**) Statistically significant correlations between Treg, naïve, and effector memory CD4+ T cells count in morbidly obese subjects with leptin, IL-10, hsCRP, and MCP-1. *P* value is significant, with a cut off value of ≤0.05 (two-tailed).

As displayed in Figure 5(A–D), one of the most important correlations observed is the significant inverse correlation between leptin, and Treg cells (r=−0.548, *p*=0.024), indicating a role of the satiety hormone in regulating the immune system (Figure 5A). Figure 5B displays a significant positive correlation between IL-10, and naïve cells (r=0.307, *p*=0.008). As shown in Figure 5C and D, hsCRP, and MCP-1 display negative relationships with TEM CD4+ cells (r=−0.261, *p*=0.025), and (r=−0.195, *p*=0.030), respectively. The limited number of study subjects impacts the limited correlations detected in the present study.

## Discussion

The current study investigated the impact of the surgical intervention by the LGS on CD4+ T regulatory cells and CD4+ T-lymphocytes subpopulations, which are: naïve, TCM, and TEM cells in the peripheral blood of class 111 obesity subjects after 12 weeks. The major finding of the study is the enhancement of the Treg cell counts by ≈1.6 folds after 12 weeks of LGS in morbidly obese subjects. Treg cells’ upsurge was supplemented by a discriminating boost of CD4+ naïve and central memory, with a parallel decrease of TEM cells and circulating leptin levels after 12 weeks of LGS. The substantial weight loss and BMI were also complemented by a substantial decrease of pro-inflammatory cytokines TNF-α, IL-6, and MCP-1, with an increase of anti-inflammatory IL-10, likely reflecting the pattern characteristic shift from Th1 to Th2 immunophenotyping polarization, from pro-inflammatory to anti-inflammatory pattern. Further, LGS reduced the frequency of metabolic syndrome (MeS) components along with deceased hsCRP associated with the improvement of leptin/adiponectin ratio. Compared to healthy control subjects, T regulatory cells showed upregulation towards healthy obese subjects but significantly lower than healthy lean subjects. IR and diabetes status has no impact on T regulatory cells before and after LGS. The implications of these results will be discussed in the next sections.

The present study demonstrated a marked inflammatory profile characteristic of obese subjects with BMI ≥ 40kg/m^2^ at the adipose tissue level and adipokines and pro-inflammatory biomarkers’ peripheral circulation. The present study demonstrated that AT of class III obesity subjects is portrayed by augmented expression of the macrophage of CD11b+ (M1 marker) by ≈8.3 folds than CD206+ (M2 marker), indicating a distinct inflammatory status. Obese subjects are characterized by AT macrophage accumulation, which may contribute to systemic insulin resistance, and local proliferation of tissue macrophages may contribute to risks of adipose tissue homeostasis and type 2 DM.^11^ Two groups of macrophages were initially defined typically as activated (M1) and alternatively activated (M2) macrophages. Macrophage infiltration in AT may be a cause and/or a consequence of the low-grade inflammatory status in obesity.^20^ The macrophage profile of class III obesity subjects in the present study agrees with previous studies demonstrating macrophage infiltration with M1 polarization in AT of obese subjects.^21^

Following the LGS, we observed a reduction of the circulating inflammatory mediators and cytokines of TNF-alpha, IL-6, MCP-1, and hsCRP, which match the significant weight loss observed. A previous study demonstrated a reduction of MCP-1 following bariatric surgery.^22^ Several studies demonstrated that hsCRP seems to be the single biomarker of inflammation observed to consistently decreased after bariatric surgery.^23^,^24^ Furthermore, the present study demonstrated a decrease of pro-inflammatory cytokines of TNF-alpha and IL-6, which is coherent with previous studies,^15^,^17^ while other studies demonstrated no changes.^18^,^19^ The inconsistent results of inflammatory cytokines profile between different studies could be due to the degree of insulin resistance at baseline, inflammatory markers’ expression, sample size, study design, technical assays, the timing of assessment of postoperative outcomes, and inherited factors of the study population, which could impact the heterogeneity among the studies.

The current data demonstrated that the anti-inflammatory markers, for instance, adiponectin and IL-10, were increased after LGS in the obese subjects, consistent with several studies that reported similar findings of these markers after bariatric surgery.^15^,^20^ A previous study by Kalra et al 2017, demonstrated that plasma leptin level increases with the fat deposition in obese subjects, and a state of leptin resistance is observed in morbidly obese subjects due to the failure of signaling to the hypothalamic satiety center.^21^ The present study demonstrated a significant reduction of leptin after 12 weeks of LGS, observed after bariatric surgery in other studies such as gastric plication, bypass, and gastric sleeve.^20^,^22^ The current study demonstrated dramatic weight loss after LGS, as previously reported.^23^ Adiponectin/leptin ratio increased in obese subjects after LGS compared to the baseline by ≈2.9 folds, indicating an improvement of adipose tissue dysfunction associated with macrophage’s polarization towards M1, insulin resistance, and alterations of adipokines, as shown in a previous study.^23^

The present study displays a comprehensive decrease in systemic inflammation coupled with a parallel decrease in fasting glucose, dyslipidemia, and insulin levels, after LGS. These changes are reflected in reduced insulin resistance as assessed by HOMA-index in the cohort Class III obese subjects after LGS. Previous studies indicated marked improvement post-bariatric surgery in alleviating hyperglycemia, hyperinsulinemia, and insulin resistance even if obesity is associated with type 2 DM.^24^,^25^ The frequency of metabolic syndrome components such as waist circumference, insulin resistance, dyslipidemia, hyperglycemia, and hypertension decreased markedly after 12 weeks of LGS among class III obesity subjects. Similar studies of follow up after LGS showed similar findings of weight loss, improvement of dyslipidemia, and metabolic syndrome.^26^ The mechanisms underlying metabolic improvement are poorly understood. These mechanisms could be caused by decreased energy intake, modification in gut hormone secretion such as peptide YY (PYY), glucagon-like peptide (GLP-1), ghrelin, bile acid action with activation of GLP-1, and change in gut microbiota activity.^29^ The above paragraphs highlighted that 12 weeks post-LGS to class 111 obese subjects could improve systemic inflammation and alleviates insulin resistance, diabetes, and metabolic syndrome.

Treg cells are a minor group of CD4+T lymphocyte populations essential for the immune system’s homeostasis, where they can manufacture IL-10 and transforming growth factor-beta (TGF-β) to control the inappropriate adaptive immune. In vascular adipose tissue (VAT) of obese mice, Treg cells had been shown to decrease compared to lean controls, highlighting its possible action in obesity-associated inflammation of adipose tissue.^30^ In experimental studies using rats on the high sucrose diet, an increase of AT’s oxidative stress was associated with decreased Treg cell functions and IL-10 secretions.^31^ Moreover, mice deficient in Treg cell in AT is susceptible to obesity linked with metabolic syndrome.^31^ Abundant resident T regulatory cells in AT was postulated to have a protective role against obesity pathogenesis via affecting the metabolism and glucose homeostasis.^10^

A previous study demonstrated a reduction of the circulating Treg cells in obese subjects and inversely correlated with inflammatory biomarkers, leptin, and BMI.^32^ Moreover, a recent study demonstrated a marked reduction of Treg lymphocytes, while CD4+ TEM cells increased in patients with morbid obesity.^33^ Children with metabolic syndrome demonstrated decreased Treg lymphocytes in the peripheral circulation.^34^ The data presented from the previous studies proposed that Treg cells play a significant function in suppressing obesity-related inflammation. The current study displayed a significant increase in Treg CD4+ lymphocytes after LGS in class 111 obese subjects compared to the pre-LGS level. Furthermore, our data indicated that post-LGS, T reg cell shifted toward healthy obese subjects, but less than lean subjects, reflecting an improvement of Treg cells.

Moreover, Treg cells’ upregulation post-LGS is not related to the insulin resistance or diabetic state, as LGS caused a significant increase of Treg cells independently. These findings need further analysis with a large sample size to confirm its correlation with IR and diabetic state, as the sample size is limited. A previous study showed that weight loss by 31.2% was observed after 16 weeks of laparoscopic greater curvature plication in class 111 obesity subjects associated with parallel decreases in leptin and circulating immune cells CD4+ and CD8+ T lymphocytes compared to their preoperative values.^22^ These data parallel the current study of decreased BMI by 32.6%, leptin, and CD4+ T lymphocytes. These circulatory changes of Treg cell number could mirror changes in the microenvironment in VAT, associated with marked loss of body weight and inflammatory status in class 111 obesity subjects.

Human blood CD4^+^ T lymphocytes are categorized into naïve CD45RA^+^ cells and memory CD45RO^+^ cells.^35^ Moreover, CD27 is expressed by all naïve CD4^+^ T cells and by nearly 80% of the CD4^+^ memory population. In the present study, bariatric surgery provoked changes in the T-cell differentiation. In our study, LGS weight loss increased naïve cell count, linked with a boost in less-differentiated TCM cells but decreased in more differentiated TEM cells. A recent study demonstrated that bariatric surgery caused alteration in memory cells with increased TCM and decreased EM cells.^36^ Naïve T cells can react to innovative pathogens that the immune system has not yet exposed, while memory cells can memorize pathogens and enhance the immune response. Naïve T cells can differentiate into memory and antigen-presenting T cells to permit an effective secondary response to identical stimuli. CD4+ effector memory cells are tangled in the manufacture of pro-inflammatory cytokines.^37^ A previous study demonstrated a correlation between TEM cells with pro-inflammatory states such as obesity.^33^

A reduction in peripheral inflammatory markers and immune cells was observed after gastric surgery in obese subjects.^38^ In parallel with a previous study, the current study’s data demonstrated that weight loss by gastric sleeve plus diet restriction shifted T cells phenotype and activation.^14^ Moreover, the marked reduction in pro-inflammatory markers, MCP-1, TNF-alpha, and IL-6 with a parallel increase in IL-10 (anti-inflammatory) after weight loss by LGS mirrors the shift of T cell phenotype subpopulations. The shift of Treg, naïve, and memory cells after surgery reflect such changes in the inflammatory state. These data are coherent with previous results that the decrease in Th1/Th2 polarization was correlated with weight gain reduction.^38^,^47^

The mechanism by which weight loss is induced by bariatric surgery in morbid obesity to remodel the immune cells is uncertain. We investigated the correlations between CD4+T reg cells before and after LGS, as well as its subpopulations of naïve, TCM, and TEM cells with the metabolic and inflammatory markers. In the present study, leptin established a negative relationship with Treg cells at baseline, indicating the correlations between leptin and the immune system. A previous study reported that in humans, leptin negatively affected the proliferation of Foxp3+CD4+CD25+ Treg.^48^ The hsCRP is an acute-phase protein that is well known to be associated with chronic low-grade inflammation as obesity. In the present study, hsCRP is negatively correlated with TEM, which could refer to the potential role of hsCRP as a modulator of stimulating the proliferation of pro-inflammatory cells, as evident in atherosclerosis.^49^ We identified a positive relationship between naïve cells and IL-10 and a negative correlation between naïve cells and MCP-1 at baseline. IL-10 is a cytokine with numerous impacts on immune system instructions and inflammation. IL-10 can impede the production of pro-inflammatory cytokines such as IFN-γ, IL-2, IL-3, and TNF-α produced by cells such as Treg cells, thus defend against tissue inflammation and damage.^50^ MCP-1 or CCL2 is the main monocyte chemoattractant in vivo and is a major chemotactic and activating factor of inflammation-associated cells such as monocytes/macrophages. The negative correlation with naïve cells could impact its proliferation and differentiation functions towards Th2 polarization.^51^

## Limitations

The current study has several limitations, such as the lack of a control group undertaking energy restriction by diet alone without surgical intervention to cause weight loss. The study’s nature as follow up, the sample size is limited, and lack of studies addressing the role of other T-lymphocytes subset such as CD8+ T lymphocytes. Lack of analysis of CD4+ and CD8+ T lymphocytes and their subtypes in adipose tissue of morbidly obese subjects is one of the major limitations. Further evaluation of AT biopsy post-surgery is aanother limitation.

To summarize, the above data indicate that LGS effectively reduces body weight as well as improved abnormal metabolic features such as hyperglycemia, dyslipidemia, and reduces the frequency of cardiovascular manifestations such as hypertension and insulin resistance. The study revealed that LGS for morbidly obese subjects increased the Treg, naïve, and TCM of CD4+ T-lymphocytes and decreased CD4+ effector memory T-cells. These changes could impact the metabolic, hormonal, and inflammatory status associated with weight loss by LGS.

## Conclusions

This pilot study realized that weight loss after gastric sleeve in class 111 obese subjects provokes remarkable shifts in immune cell profile, with a subsequent tendency towards the anti-inflammatory with cellular immune equilibrium. Our findings indicated that both the immune systems are creditable to further examination for their functions in controlling the metabolism and chronic inflammation associated with morbid obesity. It also could add knowledge to this area, which is of particular interest considering the actual pandemic of Covid-19 that put subjects with obesity at a high-risk for severe disease.

## Abbreviations

CD, cluster of differentiation; IL, interleukin; TNF-α, tumor necrosis factor; MCP, monocyte chemoattractant protein; LGS, laparoscopic gastric sleeve; HMC, Hamad Medical Corporation; TEM, effector memory; TCM, central memory; BMI, Body mass index; VAT, visceral adipose tissue; Treg, regulatory T; AT, adipose tissue; CCL5, chemokine ligand 5; ADA, American Diabetes Associaion ; QU, Qatar University; IRB, Institutional Review Board; EDTA, ethylenediaminetetraacetic acid; TLC, total leukocyte count; DLC, differential leukocyte count; BRC, Biomedical Research Center; RT, room temperature; RPM, revolutions per minute; FBS, Fetal bovine serum; SV, Stromal-Vascular; RBC, red blood cells; AST, aspartate transaminase; ALT, alanine transaminase; Type 2 DM, type 2 diabetes mellitus; WC, waist circumference; IR, insulin resistance; MeS, metabolic syndrome; HbA1C, hemoglobin A1c; TG, triglycerides; LDL-C, low-density lipoprotein cholesterol; HDL-C, high-density lipoprotein cholesterol; BW, body weight; HOMA-IR, homeostatic model assessment of insulin resistance; SBP, systolic blood pressure; DBP, diastolic blood pressure; PMNL, Polymorphonuclear leukocytes; hsCRP, high-sensitivity C-reactive protein; PYY, peptide YY; GLP-1, glucagon-like peptide; TGF-β, transforming growth factor-beta.
